# A Case of Gastric Outlet Obstruction Secondary to Parastomal Stomach Herniation

**DOI:** 10.7759/cureus.23536

**Published:** 2022-03-27

**Authors:** Sohaib Khan, Mudassir Khan, Michael Harris, Stephanie R Murphy, Paula Dionisio

**Affiliations:** 1 Internal Medicine, Parkview Medical Center, Pueblo, USA; 2 Internal Medicine, Dow University of Health Sciences, Civil Hospital Karachi, Karachi, PAK; 3 Gastroenterology, Parkview Medical Center, Pueblo, USA

**Keywords:** ileostomy, abdominal binder, ostomy belt, ng decompression, sigmoidectomy, parastomal hernia

## Abstract

Parastomal hernia (PH) is an abnormal herniation of tissue or intra-abdominal organ through the fascial defect created at the ostomy site. It is a common complication of stoma creation and usually contains bowel, intra-abdominal fat, or omentum. Herniation of a fixed organ like the stomach is very rare and can lead to significant morbidity and mortality. Here, we present a case of an 83-year-old female with a history of sigmoidectomy and subsequent development of parastomal hernia who presented with abdominal pain, nausea, and vomiting and was found to have stomach herniation into the parastomal hernia sac. She was managed conservatively with intravenous (IV) fluids, electrolyte replacement, and decompression with a nasogastric (NG) tube. In this article, we have discussed the incidence, clinical presentation, diagnosis, and management of gastric involvement in the parastomal hernia that can help clinicians identify and treat it early at the time of presentation.

## Introduction

Although there have been recent advancements in surgical practices and techniques, the incidence of parastomal hernia (PH), along with its complications, has been on the rise [1]. A parastomal hernia (PH) is an abnormal herniation of tissue or an intrabdominal organ through the fascial defect formed at an ostomy site, with the highest incidence after end colostomy at a rate of 4-48.1% [2,3]. The herniation is due to the continuous stretching of the abdominal wall by any force applied to the circumference of the ostomy [4]. Normally, a PH involves the small and large bowel, mobile and reducible omentum segments, and intra-abdominal fat [2]. Risk factors involved in the development of parastomal hernias include obesity, age, poor nutritional status, corticosteroids, malignancy, emergent procedures, and surgical techniques [5]. Stomach herniation in the parastomal sac is very uncommon and is used as an important and unusual differential diagnosis of gastric outlet obstruction [2,6]. A gastric parastomal hernia (GPH) may lead to symptoms of gastric outlet obstruction and, if incarcerated, can become a surgical emergency [3-5]. Diagnosis is made with the help of a computerized tomography (CT) scan of the abdomen and upper endoscopy to rule out pyloric obstruction [3]. Management is initially conservative and is followed by surgery to correct the defect [3]. Here, we present a case of an 83-year-old female who presented with a clinical picture of gastric outlet obstruction and was subsequently diagnosed with a gastric parastomal hernia.

## Case presentation

**Figure 1:**
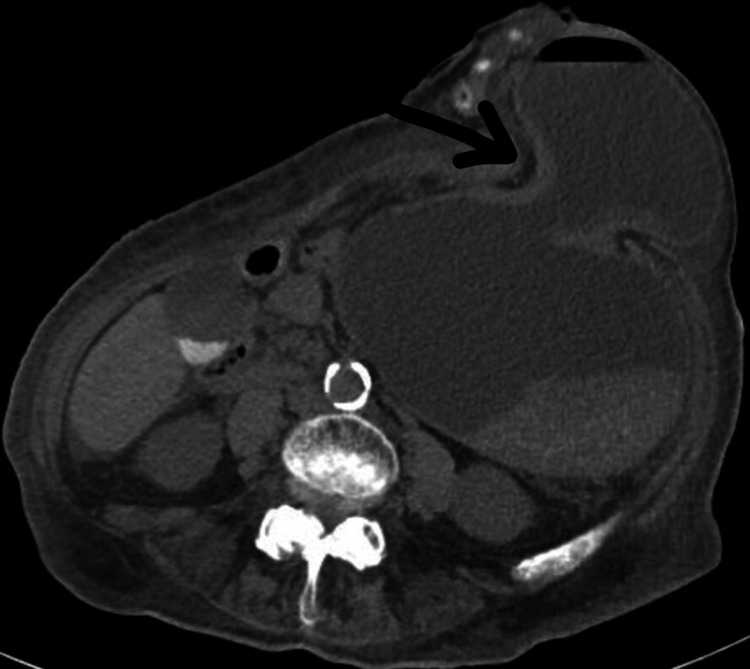
CT abdomen and pelvis on day one. Arrow showing parastomal hernia containing a portion of the distal stomach. CT: computerized tomography.

**Figure 2:**
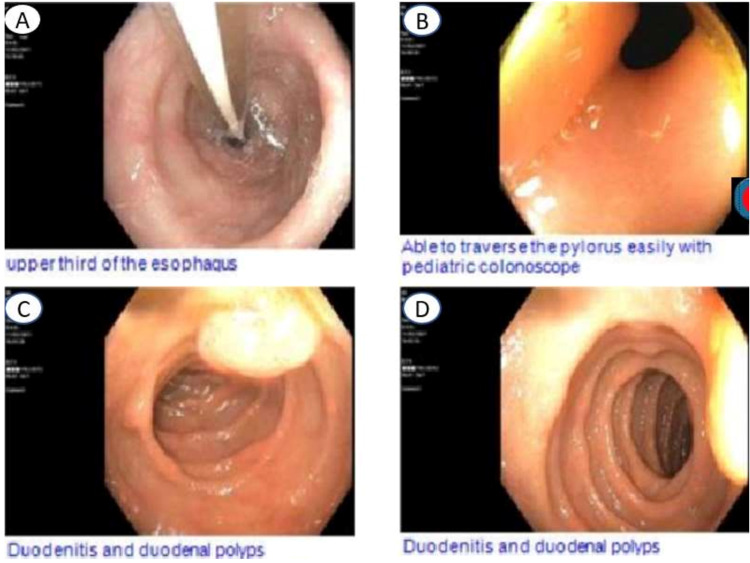
EGD shows scope that is able to traverse the pylorus, excluding intraluminal causes of obstruction. EGD: esophagogastroduodenoscopy.

**Figure 3:**
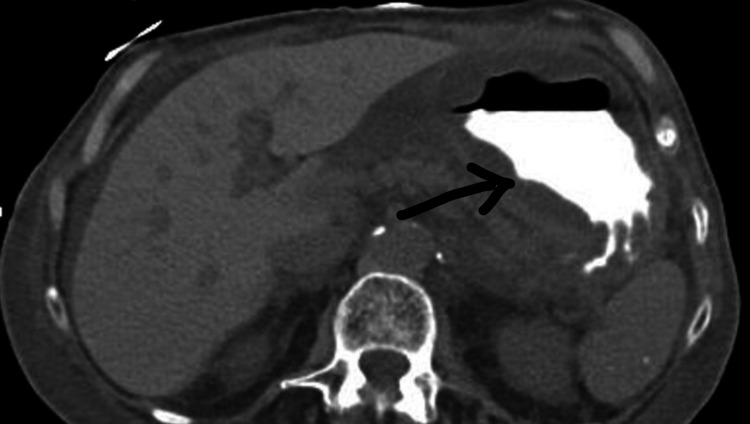
CT abdomen and pelvis on day three. Arrow showing gastric herniation and rotation into the patient’s parastomal hernia has been reduced. The stomach also looks decompressed. CT: computerized tomography.

An 83-year-old female patient, with a history of sigmoidectomy, presented with two weeks of abdominal pain. The patient previously had a large, highly vascular 2 cm polyp in the recto-sigmoid region that lead to a sigmoidectomy with an end colostomy approximately two years prior to admission. After surgery, she developed a parastomal hernia and was being followed by surgery in the outpatient setting. The abdominal pain was gradual in onset, non-radiating, located around the site of hernia, 5/10 in intensity, and was associated with abdominal distention, nausea, vomiting, and large volume output from her colostomy. At the time of admission, vitals were as follows: temperature 97.4 F, pulse 91/minutes, blood pressure 186/111 mmHg, respiratory rate 18/minutes, and saturation 97% on room air. On examination, the patient was in mild distress. An abdominal examination demonstrated a colostomy bag in the left lower quadrant with an underlying large, soft, fluctuant mass without any discharge from the ostomy bag. No tenderness to palpation, guarding, rebound tenderness, or rigidity was noted. Initial laboratory studies were significant for white cell count 7.8, hemoglobin/hematocrit 12.1/36.8, platelet count 257, sodium 145, potassium 3.2, chloride 101, bicarbonate 30, blood urea nitrogen 51, and creatinine 3.73. A computerized tomography (CT) abdomen and pelvis showed a parastomal hernia containing a portion of the distal stomach and signs of associated gastric outlet obstruction with severe gastric dilation (Figure 1). A nasogastric (NG) tube was placed for gastric decompression and the patient was started on intravenous (IV) fluids and antiemetics. General surgery and gastroenterology were consulted for further recommendations. An esophagogastroduodenoscopy (EGD) was performed, which showed esophageal food contents, duodenitis, and an ulcer in the gastroesophageal junction without any evidence of intraluminal obstruction, as the scope easily traversed through the pylorus (Figure 2A-2D). General surgery is recommended to continue NG decompression and symptomatic management. The patient was continued on a nothing per oral (NPO) diet, strict vital and input/output (I/O) monitoring, IV fluids, and NG with low intermittent wall suction. Electrolytes were continuously monitored and replaced accordingly. A repeat CT abdomen and pelvis performed on day three of admission showed an incomplete reduction of the gastric parastomal herniation along with decompression of the stomach by the NG tube (Figure 3). There was also still a loop of nondilated transverse colon extending into the hernia sac. With continued NG tube decompression and improvement of symptoms, the NG tube was removed, and the patient was started on a clear liquid diet with advancement as tolerated. On day eight of her admission, she was discharged to home with recommendations of close outpatient follow-up. 

## Discussion

Parastomal hernia (PH) is an abnormal herniation of tissue or an intra-abdominal organ through the fascial defect formed at the ostomy site [2]. It is a common complication of stoma creation with a high incidence of approximately 50% based on the type of ostomy formed [2,3,7]. It can affect 1.8-28.3% of end ileostomies, 0-6.2% of loop ileostomies, 0-38.8% of loop colostomies and as seen in our patient, 4-48% of end colostomies [2,3,6]. Usually, the PH contains bowel, intra-abdominal fat, or omentum. Very rarely, as in our case, it can involve a fixed organ like the stomach or gallbladder [2,6]. Gastric parastomal hernia (GPH) is very rare and only 19 cases were found during a literature search. It usually involves elderly patients with a female to male ratio of 13:2 and an average age of 76 years. Most of these patients have end colostomies created within the last 10 years [8-10].

GPH is extremely uncommon, in part due to the stomach’s unique intra-abdominal and anatomically secure location. It is surrounded by the diaphragm, duodenum, esophagus, liver, spleen, and transverse colon and is reinforced by multiple ligaments that provide support, integrity and prevent its herniation [1-3]. It has been postulated that increasing age and elevated pressure on a previously existing fascial defect can cause stretching and elongation of these ligament attachments. With time, constant stress and intra-abdominal pressure lead to enlargement of the fascial defect that, along with ligament laxity, leads to stomach herniation into the hernia sac [1-3,5,10].

There are numerous risk factors for GPH. These can be categorized as patient-related, technical, and surgical risk factors. The patient-related risks include female gender, malignancy, infection, chronic obstructive pulmonary disease (COPD) (causing hyperinflation and increased intra-abdominal pressure), emergent procedures, advanced age, obesity, malnutrition, use of immunosuppressive drugs, and diabetes mellitus. The technical factors include the size and type of the stoma, with an increased incidence being stated in end stoma colostomies as compared to loop stomas and ileostomies. There was also a higher incidence in stomas >25 mm as compared to small ones [1,2,6,7,10]. The risk factors present in our patient's more advanced age, female gender, formation of end colostomy, and COPD with a 30-pack-year smoking history, all of which could have contributed to the herniation of the stomach into the parastomal hernia sac.

Although mostly asymptomatic, PH carries an increased risk of becoming incarcerated or strangulated leading to perforation, gangrene, or obstruction of the organ involved. The clinical manifestation of GPH depends on the extent of gastric involvement. Most studies have shown either partial or complete gastric outlet obstruction as the presenting complaint, with symptoms ranging from abdominal pain, nausea, vomiting to strangulation and perforation [1-3]. In this case, the patient presented with abdominal pain and swelling, distention, nausea, and pain around the hernia site and had stable vitals at the time of presentation, suggesting a partial obstruction of the stomach.

GPH is usually diagnosed with a good history and physical examination. Imaging studies, including plain films with contrast or abdominal computerized tomography (CT) scans, are often utilized to identify the severity, location, contents, and complications of GPH, including perforation. Some case studies also mentioned the use of imaging studies, including fluoroscopic examination of the upper gastrointestinal tract (GI) tract to diagnose gastric parastomal hernia [2,3]. As in our case, an upper endoscopy can be performed to assist in diagnosis by ruling out other causes of gastric outlet obstruction. However, care should be taken as an incarcerated GPH could be prone to perforation from endoscopic instrumentation and, in more severe circumstances, surgical management would be preferred.

The management of GPH depends on its severity. With mild symptoms or patients with partial gastric outlet obstruction, treatments usually include nasogastric (NG) decompression, serial abdominal exams, and stoma care using an ostomy belt or abdominal binder. In more severe cases, initial management should include NG decompression followed by surgery. Indications for surgery include failure of conservative management, persistent symptoms including severe pain, distention, nausea, vomiting, irreducible bulge, obstipation, or if the patient manifests life-threatening conditions including gastric perforation, incarcerated or strangulated hernia, gastric emphysema, or septicemia. Surgical management includes stoma relocation, ostomy reversal with mesh repair for better tissue repair. Many studies have also shown a reduced incidence of parastomal hernia with prophylactic mesh placement at the time of first stoma creation [2,3,5-7,10].

## Conclusions

This case highlights the importance of gastric parastomal hernia (GPH), which is a very rare phenomenon, mostly seen in elderly females in their seventh to eighth decade of life who had end colostomies created within 10 years. The risk of GPH increases with female gender, malignancy, chronic obstructive pulmonary disease, advanced age and end colostomies, most of which were also present in our patient. Clinical presentation of GPH can range from being asymptomatic to causing vomiting, nausea, abdominal pain, strangulation, incarceration, and perforation. They are usually diagnosed with a good history and physical exam along with the computerized tomography (CT) scan and an upper endoscopy to rule out intraluminal causes of gastric outlet obstruction. Treatment of GPH includes conservative management with nasogastric (NG) decompression, serial abdominal exams, and stoma care, followed by surgery for persistent and worsening symptoms.
